# The Influence of Thermal Alterations on Prefrontal Cortex Activation and Neuromuscular Function during a Fatiguing Task

**DOI:** 10.3390/ijerph17197194

**Published:** 2020-10-01

**Authors:** Kevin Cyle Phillips, Derek Verbrigghe, Alex Gabe, Brittany Jauquet, Claire Eischer, Tejin Yoon

**Affiliations:** 1 Department of Kinesiology and Integrative Physiology, Michigan Technological University, 1400 Townsend Dr, Houghton, MI 49931, USA; drverbri@mtu.edu (D.V.); ajgabe@mtu.edu (A.G.); bacharle@mtu.edu (B.J.); cleische@mtu.edu (C.E.); 2 Department of Physical Education, Kangwon National University, 1 Gangwondaehak-gil, Hyoja-dong, Chuncheon-si, Gangwon-do 24341, Korea; tyoon@kangwon.ac.kr

**Keywords:** fNIRS, pain, RPE, muscle fatigue, brain, perception

## Abstract

The purpose of this study was to examine prefrontal cortex (PFC) activation, neuromuscular function, and perceptual measures in response to a fatiguing task, following thermal alterations of an exercising arm. Nineteen healthy adults completed three experimental sessions. At baseline, participants performed maximum voluntary isometric contractions (MVIC) of the elbow flexors. Next, participants submerged their right arm in a water bath for 15 min. Cold (C), neutral (N), and hot (H) water temperatures were maintained at 8, 33, and 44 °C, respectively. Following water immersion, participants performed an isometric elbow flexion contraction, at 20% of their MVIC, for 5 min. Ratings of perceived exertion (RPE), muscular discomfort, and task demands were assessed. Functional near-infrared spectroscopy was used to measure activation (oxygenation) of the PFC during the fatiguing task. Reductions in MVIC torque at the end of the fatiguing task were greater for the H (25.7 ± 8.4%) and N (22.2 ± 9.6%) conditions, compared to the C condition (17.5 ± 8.9%, *p* < 0.05). The increase in oxygenation of the PFC was greater for the H (13.3 ± 4.9 μmol/L) and N (12.4 ± 4.4 μmol/L) conditions, compared to the C condition (10.3 ± 3.8 μmol/L, *p* < 0.001) at the end of the fatiguing task. The increase in RPE, muscular discomfort, and task demands were greater in the H condition compared to the N and C conditions (*p* < 0.01). These results indicate that precooling an exercising arm attenuates the rise in PFC activation, muscle fatigue, and psychological rating during a fatiguing task.

## 1. Introduction

Athletes and clinical populations commonly use thermal interventions, such as cold water immersion, for preparation or recovery from fatiguing exercise [1]. Muscular fatigue is classically defined as a reversible exercise-induced reduction in force or power [2] and may originate peripherally or centrally. Peripheral fatigue occurs due to intramuscular factors at or distal to the neuromuscular junction [3], whereas central fatigue occurs proximal to the neuromuscular junction within the spinal cord or the brain [4]. More specifically, central fatigue is quantified as the reduction in voluntary activation (VA), which is the amount of neural drive used to activate skeletal muscle [2]. Despite numerous studies exploring thermal effects on mechanisms of fatigue, little research has investigated how preparatory or recovery strategies impact supraspinal cortices during exercise.

Supraspinal fatigue, a component of central fatigue, results from suboptimal output from the motor cortex [5] and can be influenced by thermal alterations. For example, Cahill and Kalmar [6] showed a reduction in central fatigue (i.e., VA quantified by magnetic stimulation of the motor cortex), following induced hypothermia, during sustained maximal contractions of the elbow flexor muscles. Conversely, passively induced hyperthermia has shown an increase in central fatigue of the elbow flexors [7]. Despite these findings, the origin and mechanisms of cold induced attenuations in central fatigue are speculative.

One major proposed mechanism of reduced VA following cooling has been a reduction in metaboreceptive afferent feedback from peripheral locations (i.e., muscle or skin) [6,8,9]. The notion that afferent feedback can alter VA suggests a conscious recognition of muscular discomfort or pain, resulting in voluntarily slowing or ceasing exercise due to an individual’s limit of “sensory tolerance” [10]. Recently, Robertson and Marino [11] proposed that the prefrontal cortex (PFC) may interpret afferent feedback and play a role in tolerating or terminating fatiguing exercise. During submaximal unilateral contractions, as the intensity of exercise increases, there is an increase in oxygenation of the PFC, as measured by functional near infrared spectroscopy (fNIRS) [12,13]. Generally, increasing the intensity or duration of resistance exercise results in metabolite accumulation which stimulates metaboreceptive muscle afferents. Additionally, thermosensitive afferents within the skin and muscle detect changes in temperature which can affect homeostasis and the sensation of discomfort [14]. Therefore, the observed increase in activation of the PFC during exercise may demonstrate the need to engage the PFC to deal with the emerging displeasure of intensifying afferent feedback [15].

To our knowledge, no study has reported PFC activation during a fatiguing task following thermal alterations of an exercising limb. Therefore, the current study aimed to examine the effects of pre-exercise temperature alterations on PFC oxygenation, during a low intensity fatiguing task. Additionally, neuromuscular function and perceptual measures were monitored. We hypothesized that cooling the exercising arm before the fatiguing task, compared to heating, would cause: (1) an attenuated rise in PFC oxygenation; (2) decreased neuromuscular fatigue and increased force steadiness; and (3) lower perceived exertion, muscular discomfort and perceived task demand.

## 2. Materials and Methods

### 2.1. Participants

Nineteen physically active young adults (17 males and 2 females) from the local university were recruited for this study. Each participant was given written and verbal explanations of the experimental procedures followed by completion of an informed consent document. Participants were excluded from this study if they had any history of muscular, neurological, or cardiovascular disease. Participants’ body mass and body fat were analyzed using bioimpedance analysis (BC-418; Tanita Corp., Tokyo, Japan). Their (mean ± SD) age, height, body mass, and body fat were 22.6 ± 3.7 years, 177.1 ± 8.7 cm, 83.0 ± 15.8 kg, and 17.1 ± 10.6%, respectively. All participants self-reported that they performed resistance exercise ≥2 days/week, however none of the participants were varsity athletes at the university. Participants were asked to refrain from alcohol and exercise of the upper body 24 h prior to each session. Additionally, participants were asked to maintain their normal caffeine intake throughout all experimental conditions. This study was approved by the Institutional Review Board at Michigan Technological University (Ethical code: M1752, (1218772-3)).

### 2.2. Study Overview

This research study included one familiarization session and three experimental sessions separated by at least 48 h. All laboratory visits were performed at approximately the same time of day, in a thermoneutral environment (23 °C). Participants were required to wear shorts, a T-shirt, and shoes for both experimental sessions. The first session involved familiarization with all equipment and testing procedures. Participants were seated and secured into the testing chair (System 4 Pro; Biodex Medical System, Shirley, NY, USA) with two nylon straps placed vertically over each shoulder and waist to restrain the participant and minimize movement during testing. All chair specifications were recorded and used for each experimental session. Following set up, participants practiced several maximal voluntary isometric contractions (MVIC) of the elbow flexors along with the fatiguing task.

**Figure 1:**
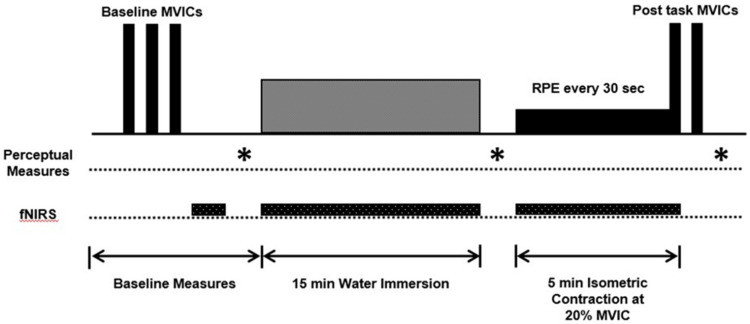
Experimental protocol.

During the three experimental sessions, participants first performed at least 3 baseline MVICs of the elbow flexors until two of the highest torque values were within 5% of each other. Next, participants submerged their exercising arm into one of the three water temperature conditions for 15 min. Before testing, six different possible temperature condition orders were determined, and each participant was randomly assigned one of the six conditions. After water immersion, participants performed a sustained isometric elbow flexion contraction at 20% of the MVIC for 5 min. Each participant was asked to trace the horizontal line with the force signal as accurately as possible, for the duration of the task. Neuromuscular function and perceptual measures were assessed throughout the experiment. fNIRS was used to examine PFC hemodynamics during the fatiguing task. An overview of the experimental protocol is shown in Figure 1.

### 2.3. Temperature Alterations and Measurements

During each experimental session, participants underwent one of three 15-min preparatory interventions before performing the fatiguing task. The exercising arm was submerged in a water bath up to the midline of the biceps brachii muscle belly and to the styloid process of the ulna, while the hand was kept out of the water. Cold, neutral, and hot water temperatures were maintained at 8, 33, and 44 °C, respectively. These temperatures were chosen based on recent research [9] and pilot testing which revealed that the 15-min duration altered skin temperature and thermal sensation.

Skin temperature was measured with an infrared thermometer (eT650D; Enno Logic, Eugene, OR, USA) at two sites directly around the electromyography (EMG) electrodes on the forearm and biceps brachii and mean values were calculated. Tympanic membrane temperature measurements were taken with a thermometer (Braun Thermoscan 5; Kaz, Marlborough, MA, USA) as a measure of core temperature. Temperature measurements were taken pre-water immersion (pre-WI), post-water immersion (post-WI) and immediately following the fatiguing task.

### 2.4. Neuromuscular Function

Participants were seated in an upright position with the right arm slightly abducted and the elbow resting on a support. Their hand was secured in a modified wrist-hand-thumb orthosis (Corflex, Manchester, NH, USA) and the forearm was positioned midway between pronation and supination. The elbow joint was flexed to 90° so that the force at the wrist was directed upwards during the MVIC. The orthosis was attached to a metal U-bolt that was attached to a transducer (SML-300; Interface, Scottsdale, AZ, USA), mounted on an adjustable support. The force data were recorded online using a Micro 1401 A-D converter and Spike2 software (CED, Cambridge, UK). The right arm of all participants was tested (two participants reported being left-hand dominant). The force exerted was displayed on a 1.77-m monitor (Sharp Electronics, Mahwah, NJ, USA) 1.5 m in front of the participant.

At the start of each experimental session, participants performed no less than three MVIC trials of the elbow flexors, followed by two MVIC trials with the elbow extensors. Participants were asked to perform elbow flexion “as hard and as fast as you can” for ~3 s. There was a 1-min rest between MVIC trials at baseline. When the peak forces from two of the three trials were not within 5% of each other, additional trials were performed until this was accomplished. The measurement of MVICs using this equipment in our laboratory was reliable across testing sessions (ICC = 0.99; 95% CI 0.98–0.99). The greatest torque achieved by the participant was taken as the MVIC torque and used as the reference to calculate the target level for the submaximal sustained fatiguing contraction of the elbow flexor muscles. Immediately following the 5-min fatiguing task, without any interruption in the contraction, participants performed an MVIC to assess neuromuscular function. This method was chosen because brief periods of relaxation before measuring the MVIC leads to an underestimation of force loss at task failure [16]. Neuromuscular function was assessed again at 20 s post task.

During each MVIC, an electrical stimulus (singlet, square wave, 100-µs duration) was used to evoke a superimposed twitch over the biceps brachii muscle at the peak torque level in the MVIC, followed by a potentiated resting twitch stimulation, approximately 2 s following the MVIC. The stimulating cathode was placed over the biceps brachii (midway between the anterior edge of the deltoid and antecubital fossa) and an anode was placed over the bicipital tendon (2 cm proximal to the elbow) [7]. Activation of the muscle was achieved by a constant-current stimulator (D185; Digitimer, Welwyn Garden City, UK). The stimulation intensity was set to 20% above the level required to produce a resting twitch of maximal amplitude so that the level of stimulation was supramaximal. This level of stimulation was used for the remainder of the protocol.

### 2.5. Electrical Recordings

At the start of each experimental session, the participants forearm was shaved, abraded, and cleaned with alcohol for surface EMG electrodes (Bagnoli DE-2.1; Delsys, Natick, MA, USA). An EMG electrode was placed on the biceps brachii, positioned lengthwise over the middle of the muscle belly. An EMG electrode was placed on the brachioradialis, at 1/3 of the distance between the styloid process of the radius and the cubital fossa, proximally. Lastly, an EMG electrode was placed on the lateral head of the triceps brachii, placed at 50% on the line between the posterior aspect of the acromion and the olecranon at two finger widths lateral to the line. A reference electrode was placed over the lateral epicondyle of the humerus. EMG recordings obtained during baseline MVICs were used to normalize subsequent activity throughout testing as a percent of the MVIC. The EMG signal was amplified (×100) and bandpass filtered at 20–450 Hz (Bagnoli-16; Delsys, Natick, MA, USA) and sampled at a rate of 2000 Hz using a Micro 1401 A-D converter and Spike2 software (CED, Cambridge, UK).

### 2.6. Prefrontal Cortex Hemodynamics

Throughout the experiment, PFC hemodynamics were monitored using an fNIRS functional brain imaging system (100 fNIRS system; fNIRS Devices LLC, Photomac, MD, USA). Sixteen optodes recorded the hemodynamics at a frequency of 2 Hz with a 2.5-cm source–detector separation. This system uses two different wavelengths of 730 and 850 nm to calculate concentration changes in oxygenated (HbO_2_) and deoxygenated hemoglobin (HHb) (both in µmol/L) using the modified Beer–Lambert Law. COBI Studio software version 1.5 (Biopac™ systems, Goleta, CA, USA) was used for data acquisition.

Following the baseline MVICs, the fNIRS headband was centered with the nasion and secured to the participant’s forehead, just above the supraorbital ridge. This fNIRS device provides an approximate assessment of Brodmann’s areas 10 (frontopolar prefrontal cortex, rostrolateral prefrontal cortex, or anterior prefrontal cortex) and 46 (dorsolateral PFC), underlying the channels [17,18]. The headband was secured with a black headband and all lighting in the laboratory was dimmed to prevent contamination of the fNIRS signal. Before the water immersion, participants were asked to relax for approximately 2 min, and a 10-s baseline measurement was performed. During this time, the participants were instructed to sit still with their eyes open, breath normally, relax, and not control their mental activity in any particular way. Thereafter, changes in HbO_2_ and HHb concentration from this 10-s rest period baseline were calculated throughout the entire experiment. Participants were asked to minimize head and body movements and to breathe gently and regularly throughout the experiment to reduce artifact in the signal.

### 2.7. Perceptual Measures

Participants were asked to provide their rating of perceived exertion (RPE), on a scale of 0–10 according to Borg [19]. Participants were instructed to focus the assessment of effort on the exercising arm. The scale was anchored so that 0 represented a resting state and 10 corresponded to the strongest effort the participant could perform to maintain the target force line. The RPE was recorded at the start of the task and every 30 s until the end of the fatiguing task.

Participants were asked to rate their whole body and right arm thermal sensation separately, ranging from 1 (unbearably cold) to 13 (unbearably hot), with 7 representing neutral. Participants were also asked to rate their perception of thermal discomfort and right arm muscular discomfort, ranging from 1 (comfortable) to 5 (extremely uncomfortable) [7,20]. Perceptual measures were taken pre-WI, post-WI, and post exercise.

Lastly, after completing the fatiguing task in each condition, participants were asked to complete the National Aeronautics and Space Administration Task Load Index (NASA-TLX) [21], which evaluated the task demand (mental, physical, temporal, effort, performance, frustration). Numerical ratings for each subscale were obtained by having the participant mark a line (19-point scale) to represent their perception of workload from low to high.

### 2.8. Data Analysis

All torque data were analyzed using Spike2 software (CED, Cambridge, UK). The torque for the MVIC and submaximal contractions were calculated as the product of force and the distance between the elbow joint and the point at which the wrist was attached to the force transducer. The MVIC torque was quantified as the average value over a 0.5-s interval that was centered about the peak. The maximal EMG for each muscle was determined as the root mean squared (RMS) value over a 0.5-s interval about the same interval of the MVC torque measurement.

The fluctuations in force and the RMS of the EMG signals were quantified during the fatiguing contraction at the following time intervals: 0–25%, 25–50%, 50–75%, and 75–100% of the task duration. The EMG activity of the elbow flexor and extensor muscles during the fatiguing contraction was normalized to the RMS EMG value obtained during the MVC for each muscle. The amplitude of the force variability was quantified as the coefficient of variation (CV; standard deviation of the force/mean of force × 100).

Voluntary activation was assessed using the interpolated twitch technique, calculated as voluntary activation = 100 × (1–T_interpolated_/T_control_), where T_interpolated_ is the amplitude of the interpolated twitch and T_control_ is the amplitude of the resting twitch produced in the relaxed but potentiated muscle [22].

The raw fNIRS data were pre-processed using fnirSoft version 4.8 (Biopac™ systems, Goleta, CA, USA). The fNIRS data from the entire experiment were bandpass filtered using a FIR filter with an order of 20 and cutoff frequencies of 0.01 and 0.1 Hz. All data were visually inspected before converting to a MATLAB file for analysis. Optodes with clearly abnormal concentration changes due to poor skin contact were excluded. In this situation, excluded optodes were substituted by the participants mean concentration calculated from the other available optodes in that specific region (right PFC = Optodes 1–6 (right dorsolateral PFC), central PFC = Optodes 7–10 (frontopolar prefrontal cortex, rostrolateral prefrontal cortex, or anterior prefrontal cortex), left PFC = Optodes 11–16 (left dorsolateral PFC)) [17,18]. Nine participants had missing or abnormal data for at least one optode during an experiment. No participants had more than one abnormal optode in a region of interest. Out of 912 optodes ((16 optodes × 3 session) × 19 participants), 12 optodes were fixed before analysis.

The blood-oxygenation response was calculated as the difference between HbO_2_ and HHb (Oxygenation = HbO_2_ – HHb). This was used as an index of tissue oxygen extraction. For the analysis, concentration changes (relative to baseline) were analyzed as the mean value during the first and last 10 s of the fatiguing task, along with each 10-s interval before each RPE rating (i.e., 20–30 s, 50–60 s, 80–90 s, etc.). Ten-second blocks were chosen based on the response pattern of the NIRS signals and studies focusing on cortex oxygenation responses to cognitive or motor tasks [13,23,24]. There were no significant differences between the response of the right, central, and left PFC, therefore the data were reported as changes in the whole prefrontal cortex (16 optodes averaged).

### 2.9. Statistical Analysis

Separate two-way repeated measures analysis of variance (ANOVAs) were used to detect main effects of temperature condition and time (baseline, post task, and 20 s post task) on changes in MVIC torque, voluntary activation, and contractile properties. If a significant main effect of temperature condition was found, then subsequent pairwise comparisons (Fisher’s least significant difference) were used to determine the source of significance at specific time points. Separate two-way repeated measures ANOVAs were used to examine main effects of temperature condition and time (11 time points) on changes in PFC oxygenation and RPE. A one-way repeated measures ANOVA was performed to examine differences in oxygenation and RPE at the start and end of the fatiguing task. Separate two-way repeated measures ANOVA were used to detect main effects of temperature condition and time (0–25%, 25–50%, 50–75%, and 75–100% of the duration of the fatiguing task) on changes in force variability and EMG. If a significant main effect of temperature condition was found, then subsequent pairwise comparisons (Fisher’s least significant difference) were used to determine where the differences occurred. Separate two-way repeated measures ANOVAs were used to detect main effects of temperature condition and time (Pre-WI, Post-WI, and post exercise) on perceptual measures and temperatures. A one-way repeated measures ANOVA was used to detect differences in NASA-TLX scores between temperature conditions. Lastly, no sex-related differences in the main variables were found, thus we analyzed the male and female data together.

Pearson correlation coefficients (*r*) were calculated between selected pairs of variables. For each ANOVA the sphericity of data was verified with Mauchly’s test and technical corrections were performed whenever necessary. Partial eta squared (ηp^2^) was calculated as a measure of effect sizes with ηp^2^ ≥ 0.01 indicating small, ≥0.059 medium, and ≥0.138 large effects [25]. Statistical procedures were performed using SPSS 24 (Armonk, NY, USA). Data are reported as mean ± SD and alpha was set to 0.05.

## 3. Results

### 3.1. Skin and Core Temperature

All temperature data are presented in Table 1. There was a significant effect of time (F_3,16_ = 23.6, *p* < 0.001, ηp^2^ = 0.734) and temperature (F_2,17_ = 203.3, *p* < 0.001, ηp^2^ = 0.960) on skin temperature of the brachioradialis. There was a significant interaction (F_4,15_ = 223.1, *p* < 0.001, ηp^2^ = 0.983). There was a significant effect of time (F_2,17_ = 4.49, *p* = 0.027, ηp^2^ = 0.346) and temperature (F_2,17_ = 101.4, *p* < 0.001, ηp^2^ = 0.923) on skin temperature of the biceps brachii. There was a significant interaction (F_4,15_ = 50.4, *p* < 0.001, ηp^2^ = 0.931). Core temperature remained unchanged throughout the experiments.

### 3.2. MVIC Torque

**Figure 2:**
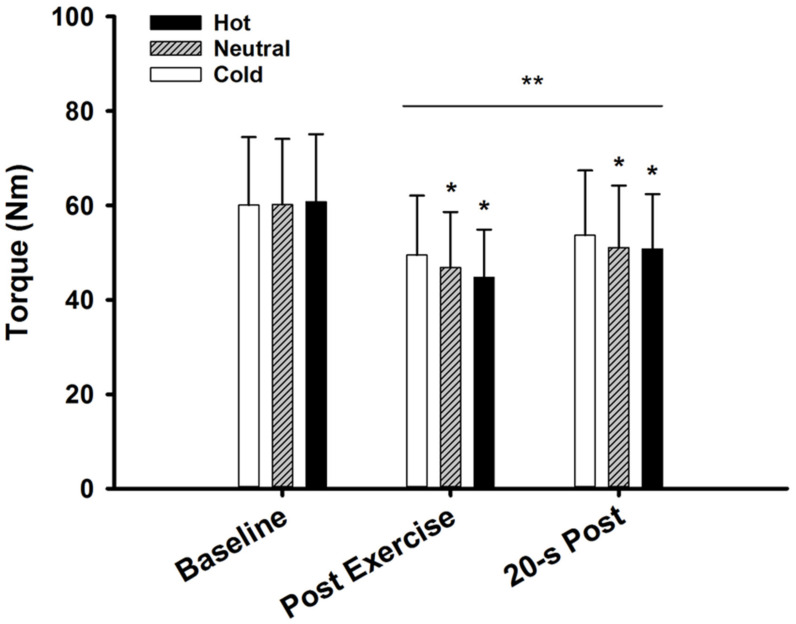
MVIC torque throughout testing (Baseline), MVIC at the end of the fatiguing task (Post Exercise), and 20 s post fatiguing task (20-s Post). * (*p* < 0.05) vs. cold. ** (*p* < 0.05) vs. baseline.

MVIC torque data are presented in Figure 2. There was a main effect of temperature (F_2,36_ = 5.72, *p* = 0.007, ηp^2^ = 0.241) and time (F_2,17_ = 50.0, *p* < 0.001, ηp^2^ = 0.855) for MVIC torque. There was a significant interaction between time and temperature (F_4,15_ = 4.90, *p* = 0.01, ηp^2^ = 0.566). At baseline, torque was similar among the cold, neutral, and hot conditions (60.1 ± 14.4 vs. 60.2 ± 13.9 vs. 60.8 ± 14.3 Nm; F_2,36_ = 0.981, *p* = 0.385). There was a significant reduction in MVIC torque at the end of the fatiguing task; however, the reduction in MVIC torque was greater for the hot (25.7 ± 8.4%) and neutral (22.2 ± 9.6%) conditions, compared to the cold condition (17.5 ± 8.9%; F_2,17_ = 6.54, *p* = 0.008, ηp^2^ = 0.435). There was a significant recovery in MVIC torque 20 s post task (time effect, F_1,18_ = 347.8, *p* < 0.001, ηp^2^ = 0.951). MVIC torque was greater in the cold condition compared to the neutral and hot condition at 20 s post task (temperature effect, F_2,36_ = 4.82, *p* = 0.014, ηp^2^ = 0.211).

### 3.3. Voluntary Activation

There was a main effect of time (F_2,17_ = 4.52, *p* = 0.027, ηp^2^ = 0.347) on voluntary activation. There was no effect of temperature (*p* = 0.211). There was no interaction (*p* = 0.523). At baseline, voluntary activation was similar among temperature conditions (*p* = 0.948). Voluntary activation measured at the end of the fatiguing task was similar among the cold, neutral, and hot condition (97.3 ± 3.5 vs. 96.7 ± 4.7 vs. 95.3 ± 6.0%; F_2,36_ = 1.71, *p* = 0.195). Voluntary activation measured 20 s after the end of the fatiguing task was similar among the cold, neutral, and hot condition (96.7 ± 3.0 vs. 95.6 ± 4.6 vs. 95.1 ± 4.4%; F_2,36_ = 1.15, *p* = 0.325).

### 3.4. Muscle Contractile Properties

There was a main effect of time (F_2,17_ = 75.4, *p* < 0.001, ηp^2^ = 0.899) on resting twitch. There was no effect of temperature (*p* = 0.458) and no interaction (*p* = 0.227). At baseline, resting twitch was similar between temperature conditions (*p* = 0.954). There was a significant reduction in resting twitch from baseline to the end of the fatiguing task, however values did not differ among temperature conditions (temperature effects, F_2,36_ = 0.68, *p* = 0.510). Resting twitch 20 s post fatiguing task was similar among temperature conditions (temperature effects, F_2,36_ = 1.61, *p* = 0.216).

There was a main effect of temperature (F_2,36_ = 11.3, *p* < 0.001, ηp^2^ = 0.387) on time to peak. There was no effect of time (*p* = 0.284). There was a significant interaction (F_4,15_ = 10.9, *p* < 0.001, ηp^2^ = 0.745). Time to peak at baseline did not differ among temperature conditions (*p* = 0.244). Time to peak at the end of the fatiguing task was quicker in the hot and neutral conditions compared to the cold condition (temperature effects, F_2,17_ = 42.1, *p* < 0.001). Time to peak 20 s post fatiguing task was quicker in the hot and neutral conditions compared to the cold condition (temperature effects, F_2,17_ = 18.9, *p* < 0.001).

There was a main effect of time (F_2,17_ = 33.9, *p* < 0.001, ηp^2^ = 0.800) and temperature (F_2,36_ = 4.13, *p* = 0.024, ηp^2^ = 0.187) on half relaxation time. There was a significant interaction (F_4,15_ = 9.71, *p* < 0.001, ηp^2^ = 0.721). Half relaxation time at baseline was similar between temperature conditions (*p* = 0.319). Half relaxation time at the end of the fatiguing task was quicker in the hot and neutral conditions compared to the cold condition (temperature effects, F_2,36_ = 6.64, *p* = 0.004). Half relaxation time 20 s post fatiguing task was quicker in the hot and neutral conditions compared to the cold condition (temperature effects, F_2,36_ = 6.53, *p* = 0.004).

### 3.5. Force Variability

**Figure A1:**
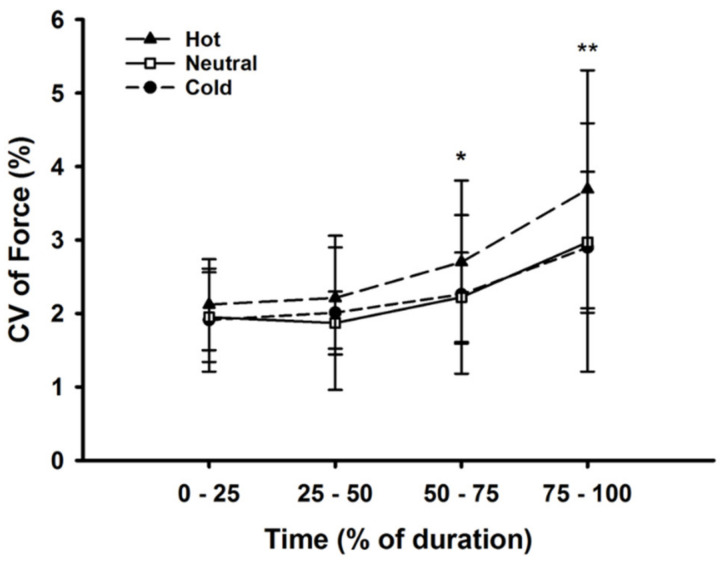
Force variability throughout the fatiguing task. * (*p* < 0.05) hot vs. cold. ** (*p* < 0.01) hot vs. neutral.

Force variability data are presented in Figure A1. There was an increase in force variability throughout the fatiguing task (Time effect, F_3,16_ = 20.7, *p* < 0.001, ηp^2^ = 0.574). A main effect of temperature was also noted (F_2,36_ = 4.25, *p* = 0.022, ηp^2^ = 0.191). There was no interaction (*p* = 0.380). During the third quarter (50–75%) of the fatiguing task duration, force variability was greater in the hot condition compared the cold and neutral conditions (*p* < 0.05). During the fourth quarter (75–100%) of the fatiguing task duration, force variability was greater in the hot condition compared to the neutral condition (*p* < 0.01).

### 3.6. Electromyography

**Figure A2:**
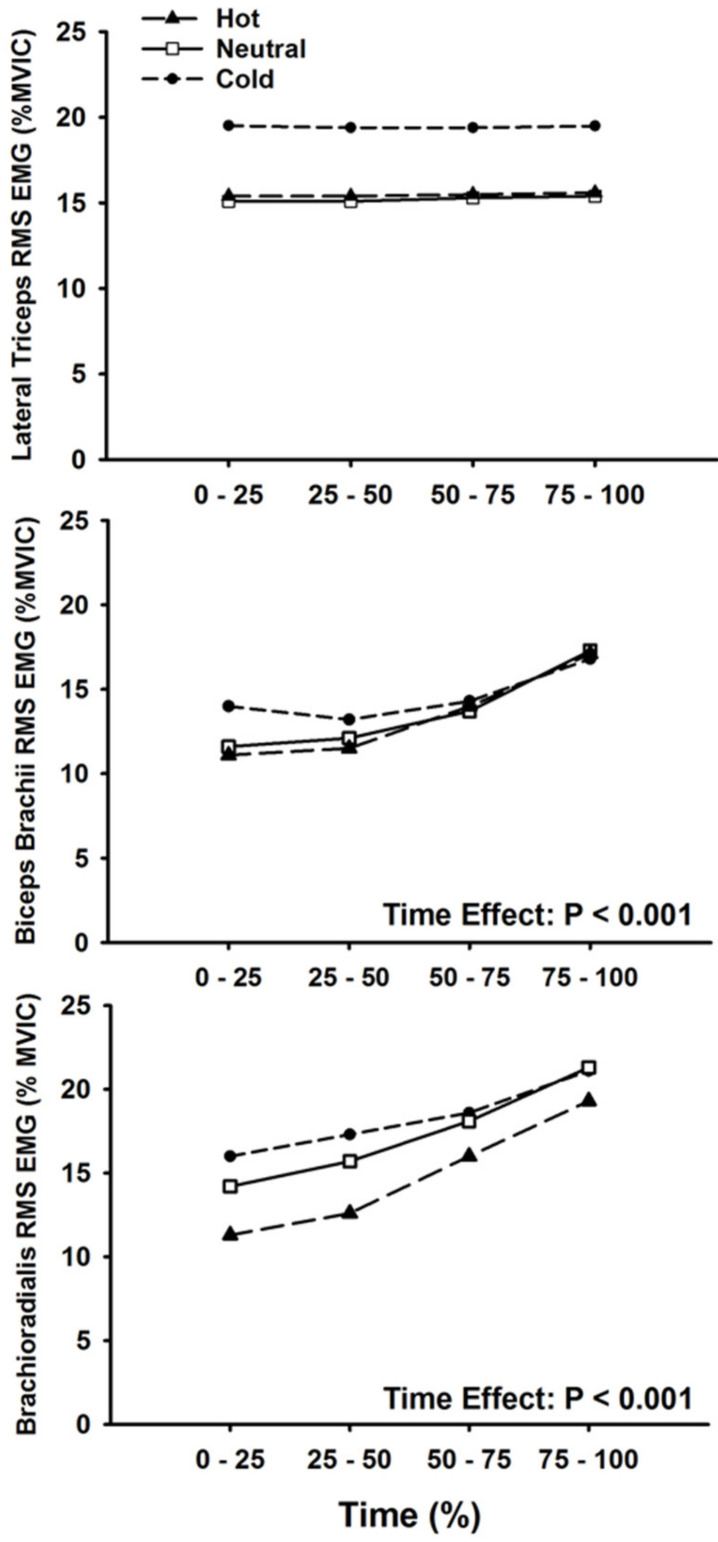
EMG throughout the fatiguing task. There was a significant increase in muscle activity of the biceps brachii and brachioradialis during the fatiguing task (time effect: *p* < 0.001). Data are reported as mean values. Standard deviation bars were removed for clarity.

Electromyography data are presented in Figure A2. There was a main effect of time (F_3,16_ = 11.7, *p* < 0.001, ηp^2^ = 0.687) on muscle activity of the brachioradialis muscle. There was no effect of temperature and no interaction. There was a main effect of time (F_3,16_ = 15.8, *p* < 0.001, ηp^2^ = 0.748) on muscle activity of the biceps brachii muscle. There was no effect of temperature and no interaction for the biceps brachii muscle. There was a main effect of time (F_3,16_ = 6.85, *p* = 0.003, ηp^2^ = 0.563) on muscle activity of the triceps brachii muscle. There was no effect of temperature and no interaction.

### 3.7. Prefrontal Cortex Oxygenation

**Figure 3:**
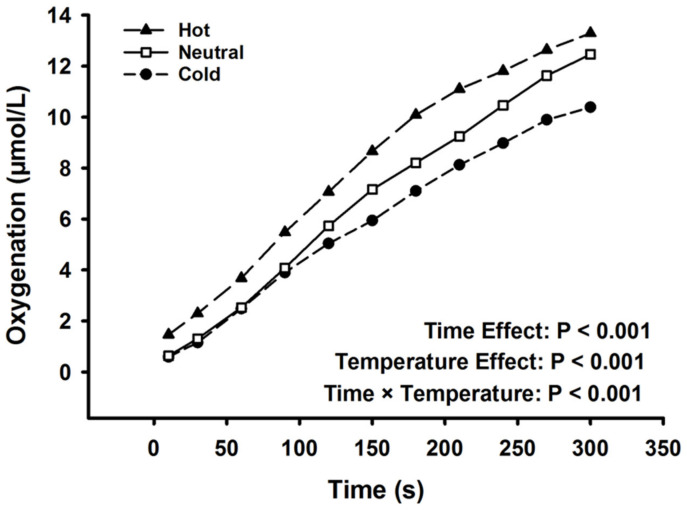
Prefrontal cortex oxygenation, as calculated as change from baseline values, throughout the fatiguing task. There was a main effect of time, temperature condition, and an interaction (*p* < 0.001). Data are displayed as mean values. Standard deviation bars were removed for clarity.

PFC oxygenation data are presented in Figure 3. Oxygenation at the start of the fatiguing task (first 10 s) was similar among the cold, neutral, and hot conditions (0.58 ± 1.9 vs. 0.63 ± 1.6 vs. 1.46 ± 2.1 μmol/L, F_2,36_ = 1.91, *p* = 0.163). There was a significant effect of time (F_10,9_ = 14.9, *p* < 0.001, ηp^2^ = 0.943) and temperature (F_2,36_ = 10.8, *p* < 0.001, ηp^2^ = 0.375) on PFC oxygenation throughout the fatiguing task. There was a significant interaction between temperature condition and time throughout the fatiguing task (F_20,360_ = 5.08, *p* < 0.001, ηp^2^ = 0.220). Oxygenation at the end of the fatiguing task (last 10 s) was greater in the hot (13.3 ± 4.5 μmol/L) and neutral conditions (12.4 ± 4.4 μmol/L), compared to the cold condition (10.3 ± 3.8 μmol/L; F_2,36_ = 10.2, *p* < 0.001, ηp^2^ = 0.362).

### 3.8. Perceived Exertion

**Figure 4:**
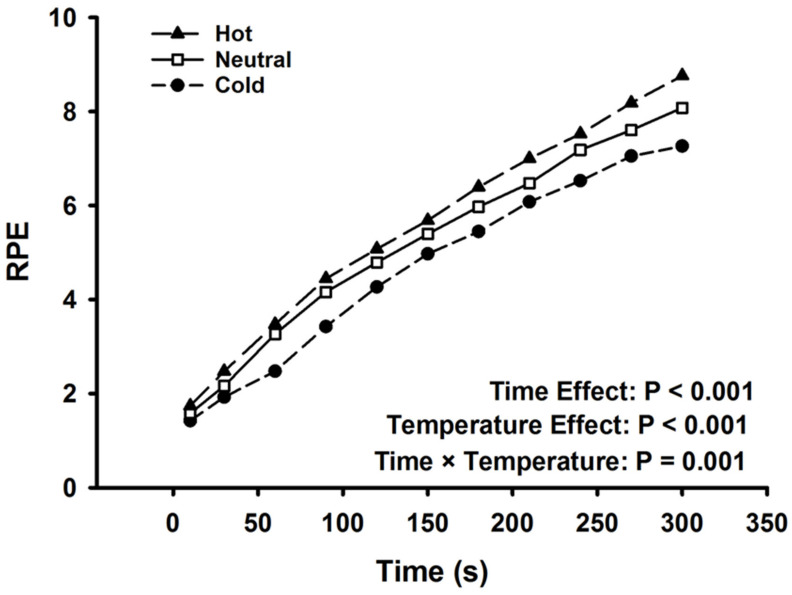
Ratings of perceived exertion throughout the fatiguing task. There was a main effect of time and temperature condition as well as an interaction (*p* < 0.001). Data are displayed as mean values. Standard deviation bars were removed for clarity.

Rating of perceived exertion data are presented in Figure 4. Participants ratings of perceived exertion did not differ among the cold, neutral, and hot condition at the start of the fatiguing task (1.4 ± 0.6 vs. 1.6 ± 0.7 vs. 1.7 ± 0.7; F_2,36_ = 2.98, *p* = 0.063). There was a significant effect of time (F_10,9_ = 94.8, *p* < 0.001, ηp^2^ = 0.991) and temperature (F_2,17_ = 12.6, *p* < 0.001, ηp^2^ = 0.599) on perceived exertion throughout the fatiguing task. There was a significant interaction between time and temperature (F_20,360_ = 2.37, *p* = 0.001, ηp^2^ = 0.116). Participants ratings of perceived exertion were greater at the end of the hot (8.7 ± 0.9) and neutral conditions (8.1 ± 0.9), compared to the cold (7.2 ± 1.1; F_2,36_ = 35.3, *p* < 0.001, ηp^2^ = 0.663).

Participants rated the overall workload of the fatiguing task as significantly greater in the hot and neutral conditions, compared to the cold condition (temperature effect, F_2,36_ = 9.43, *p* = 0.001, ηp^2^ = 0.344; Table A1).

### 3.9. Thermal Sensation and Discomfort

Thermal sensation and discomfort data are presented in Table 1. There was a significant effect of time (F_2,17_ = 9.17, *p* = 0.002, ηp^2^ = 0.519) and temperature (F_2,17_ = 13.4, *p* < 0.001, ηp^2^ = 0.613) on thermal sensation of the body. There was a significant interaction (F_4,15_ = 5.91, *p* = 0.005, ηp^2^ = 0.612). There was a significant effect of time (F_2,17_ = 17.3, *p* < 0.001, ηp^2^ = 0.671) and temperature (F_2,17_ = 100.6, *p* < 0.001, ηp^2^ = 0.922) on thermal sensation of the arm. There was a significant interaction (F_4,15_ = 306.3, *p* < 0.001, ηp^2^ = 0.988).

There was a significant effect of time (F_2,36_ = 273.5, *p* < 0.001, ηp^2^ = 0.938) and temperature (F_2,36_ = 3.58, *p* = 0.038, ηp^2^ = 0.166) on muscular discomfort of the exercising arm. There was a significant interaction (F_4,72_ = 14.7, *p* < 0.001, ηp^2^ = 0.450). There was a significant effect of time (F_2,36_ = 17.2, *p* < 0.001, ηp^2^ = 0.489) and temperature (F_2,36_ = 5.02, *p* = 0.012, ηp^2^ = 0.218) on thermal discomfort of the body. There was a significant interaction (F_4,72_ = 8.88, *p* < 0.001, ηp^2^ = 0.330).

### 3.10. Correlations

Despite the relatively small number of participants, several correlations should be mentioned. A significant correlation was noted between the loss in MVIC torque and resting twitch loss after the fatiguing task for the hot (*r* = 0.73, *p* < 0.001) and neutral conditions (*r* = 0.67, *p* < 0.01). There was a significant negative correlation between the loss in MVIC torque and the increase in PFC oxygenation during the fatiguing task in the hot (*r* = -0.68, *p* < 0.01), neutral (*r* = -0.48, *p* < 0.05), and cold conditions (*r* = -0.47, *p* < 0.05). There was a significant correlation between the increase in oxygenation of the PFC and increase in RPE during the fatiguing task in the hot condition (*r* = -0.47, *p* < 0.05).

## 4. Discussion

The aim of this study was to examine the effects of local thermal alterations on PFC oxygenation, neuromuscular function, and perceptual measures during and after a sustained submaximal isometric contraction. Our primary finding is that precooling the exercising arm caused an attenuated rise in PFC activation, muscle fatigue, force variability, perceived exertion, muscular discomfort, and ratings of task demand. Conversely, preheating had an accelerating effect on these variables. These results indicate that local changes in temperature can alter PFC activation, neuromuscular function, and perceptual demands during a low intensity fatiguing task.

### 4.1. Temperature Effects on Peripheral Fatigue

The 5-min submaximal contraction performed in the current study successfully caused muscular fatigue, as shown by the significant reductions in MVIC torque at the end of the fatiguing task. Reductions in MVIC torque at the end of the fatiguing task were greater for the hot (25.7 ± 8.4%) and neutral (22.2 ± 9.6%) conditions compared to the cold condition (17.5 ± 8.9%). The reductions in MVIC torque can partially be explained by the declines in resting twitch torque found post task [3]. However, there was no temperature effect on resting twitch torque post task. Nonetheless, there was a strong correlation between the reductions in MVIC torque and resting twitch torque in the hot and neutral conditions.

Significant temperature effects on time to peak torque and time to half relaxation were also noted. The interactions found in this study indicate that the cooled muscle had a slower time to peak torque and a longer half relaxation time compared to the neutral and hot conditions. These findings are in agreement with those of Davies and Young [26] who found that reducing muscle temperature by 8.4 °C increased time to peak torque and half relaxation time. However, they also found a reduction in resting twitch torque and MVIC torque [26]. Although direct muscle temperature was not taken in the current study, it is possible that reductions in muscle temperature were not great enough to impair resting twitch torque or MVIC torque. Regardless, reductions in contractile and relaxation speeds are beneficial for low to moderate intensity contractions because cooling a muscle decreases ATPase activity [27] and thus the accumulation of metabolic by-products which hinder Ca^2+^ kinetics and ultimately the power stroke phase of muscle fiber contractions [28].

### 4.2. Temperature Effects on Central Fatigue

Unexpectedly, the current study found no significant reductions or temperature effects on VA at the end of the fatiguing task. Whole-body hyperthermia and hypothermia causes significant declines in brief MVICs [6,7], however modifying muscle temperature over a wide range has no effect on MVIC force when contractions are brief [9]. When performing a sustained 2-min MVIC, there is a progressive reduction in VA and torque as core [7] or muscle temperature [9] increases. On the other hand, hypothermia [6] and muscle cooling [9] has an attenuating effect on VA and torque during a sustained MVIC. Lastly, although Lloyd and Hodder [9] found significant muscle temperature effects on VA during their fatiguing task, no temperature effect was noted for VA at the end of the 120-s contraction.

Indeed, low intensity contractions can cause considerable central fatigue [29,30]. However, the fatiguing task in the current study was a set duration rather than until failure. The rational for a standardized duration of 5 min was based on extensive pilot testing that showed participants could maintain the required torque level while also reaching a high level of perceived exertion. Additionally, a set duration allowed for an equal comparison between dependent variables. Although participants reached a relatively high level of perceived exertion by the end of the task, ratings did not reach a maximal level. Therefore, future research should examine temperature effects during a low intensity contraction for a longer duration.

### 4.3. The Psychophysiological Cost of Thermal Alterations

The current study found that precooling the exercising arm before performing a sustained submaximal contraction resulted in an attenuated rise in PFC oxygenation (oxygen extraction) throughout the fatiguing task, whereas preheating accelerated the rise in PFC oxygenation. Functional NIRS provides an indirect method of assessing brain activation based on the concept of neurovascular coupling, which is the tightly coupled vascular response associated with neuronal activation [31]. Therefore, our results suggest that, as fatigue developed throughout the 5-min task, there was an increased activation of the PFC in order to continue exercise, despite the emerging displeasure associated with fatiguing exercise. Moreover, thermal alterations modified the rate of change in both physiological and psychological variables.

These results provide evidence for the theory that the PFC plays a role in interpreting afferent feedback and regulating affect (i.e., pain, displeasure, and fatigue) during fatiguing tasks [11,15]. Negative affect is known to influence exercise tolerance and may play a role in the resulting response to uncomfortable sensations [32]. Several studies have shown that the amygdala is a key region of convergence for exercise-related interoceptive information [33]. Davidson [34] and Davidson [35] suggested that the PFC exerts inhibitory control over the amygdala during exposure to adverse stimuli, which helps regulate the negative affective responses. In support of this, studies have shown an increase in activation of the PFC with a concomitant attenuation of activity in the amygdala, in response to negative affect [36,37].

The role of muscle cooling decreasing afferent feedback to the PFC may partly be explained by a delayed exercise-induced increase in muscle sympathetic nerve activity following muscular cooling [8], while increasing local tissue temperature significantly increases muscle sympathetic nerve activity [38]. Cooling in the current study reduced thermal strain, as supported by lower skin temperatures and thermal sensation ratings. Additionally, all fatiguing tasks were performed at a similar intensity; however, perceptual measures revealed that precooling attenuated the rise in perceived exertion during the fatiguing task, along with ratings of muscular discomfort at the end of the fatiguing task. Moreover, there was a moderate correlation between the rise in oxygenation and perceived exertion during the hot condition throughout the fatiguing task. Finally, overall task demand, assessed by the NASA-TLX after completion of the fatiguing task, was perceived as lower during the cold condition. Therefore, altering the temperature of the exercising arm influenced the participants’ perception of fatigue and tolerance of exercise intensity [10].

To our knowledge, only two studies have examined PFC hemodynamics in response to temperature alterations. Morrison and Sleivert [39] examined the role of passive hyperthermia on PFC oxygenation during a 10-s MVIC of the knee extensors. They found that passive heating did not alter PFC oxygenation, but cooling back to baseline core temperature attenuated cerebral oxygenated and total hemoglobin levels. Minett and Duffield [40] examined the effects of post-exercise cooling on neuromuscular, physiological, and PFC hemodynamic responses after intermittent sprinting in the heat. They revealed that cold water immersion of the body hastened recovery of MVIC torque of the knee extensors and reduced PFC oxygenation during an MVIC post recovery and 1 h post recovery. The authors explained that the reduction in cerebral oxygenation and improved central nervous system drive (improved central activation ratio and RMS EMG) after cold water immersion suggested the blood volume shift to be a reflex response to greater heat removal with post-exercise cooling. Unlike the above-mentioned studies, which presented PFC data recorded during a short duration MVIC, the current study provides a robust examination of PFC oxygenation throughout an entire fatiguing task.

### 4.4. Implications for Sports, Exercise, and Physical Activity

Cold water immersion is gaining popularity as a method of preparing for exercise and accelerating post exercise recovery [1]. For example, Heyman and De Geus [41] and Baláš and Chovan [42] studied multiple methods of recovery between subsequent bouts of rock climbing until failure. Both studies found that cold water immersion of the arms preserved climbing performance in subsequent fatiguing tasks, compared to passive recovery. However, the authors were still at a loss for physiological evidence to explain the mechanisms of improved performance. The authors did however suggest that cold water immersion may have decreased the perception of fatigue. We propose that the psychophysiological benefits of cooling observed in this study could help explain these performance improvements. Additionally, the improved motor performance and reduction perceived workload demands have important implications for workers, athletes, and clinical populations participating in low intensity exercise.

### 4.5. Limitations

Several limitations should be addressed. First, although the PFC is not directly connected with major motor control regions, it is indirectly linked via the premotor area and thus may assist in exercise regulation [11]. Secondly, fNIRS provides us with an indirect measure of PFC activation and the validity of fNIRS has been compared with MRI measurements, showing close agreement between measurements [43]. However, the overall penetration depth of fNIRS is only ~2.5 cm, which is sufficient to penetrate the grey matter of cerebral tissue in adults, but these hemodynamic changes may not be representative of deeper cerebral tissue [44]. Another limitation that should be considered is that pre-testing caffeine consumption intake was not reported by participants, which may influence fatigability. Lastly, although there was no changes in core temperature post-water immersion, we cannot rule out if skin or scalp blood flow (and therefore oxyhemoglobin) were altered, which can influence cortical measurements [45].

## 5. Conclusions

Performance of an isometric contraction at 20% of MVIC for 5 min following muscle cooling attenuated the rise in PFC oxygenation, perceived exertion, and muscle fatigue. Conversely, preheating accelerated the rise in these variables. Additionally, ratings of muscular discomfort and task demand were perceived as greater in the hot condition. These results provide important implications for athletes and clinical patients performing prolonged low intensity contractions. Cold water immersion provides a simple method of decreasing negative affect associated with fatiguing contractions and improving motor performance. Future research should explore temperature effects on PFC oxygenation during full body or larger muscle mass exercise.

## Figures and Tables

**Table 1:** Temperature and perceptual measures throughout the experiment.

Variable	Pre-Water Immersion	Post-Water Immersion	Post Fatigue
Brachioradialis Skin Temp (◦C)			
Cold	32.1 ± 1.1	21.3 ± 2.7 ##	27.3 ± 2.4 ††
Neutral	31.6 ± 1.5	31.1 ± 1.1 **	31.4 ± 1.6 **
Hot	31.7 ± 1.2	36.1 ± 1.1 ## **	34.3 ± 0.6 †† **
Bicep Brachii Skin Temp (◦C)			
Cold	32.6 ± 1.1	28.5 ± 2.5 ##	31.7 ± 1.2 ††
Neutral	32.3 ± 1.4	32.4 ± 1.4 **	32.4 ± 1.4 **
Hot	32.1 ± 1.4	35.8 ± 1.1 ## **	34.2 ± 1.1 †† **
Core Temp (◦C)			
Cold	36.8 ± 1.12	36.8 ± 0.69	36.6 ± 1.38
Neutral	36.8 ± 0.43	36.7 ± 0.40	36.5 ± 0.53
Hot	36.7 ± 0.42	36.8 ± 0.34	36.8 ± 1.3
Thermal Sensation (Body)			
Cold	6.94 ± 0.22	6.02 ± 0.88 ##	7.13 ± 0.77 ††
Neutral	7.21 ± 0.71	7.13 ± 0.32 **	7.65 ± 0.81 † *
Hot	7.05 ± 0.22	8.26 ± 1.19 † **	8.39 ± 1.38 **
Thermal Sensation (Arm)			
Cold	7.05 ± 0.22	3.18 ± 0.61 ##	5.81 ± 0.93 ††
Neutral	7.05 ± 0.40	7.02 ± 0.35 **	7.92 ± 1.05 † **
Hot	7.00 ± 0.33	9.68 ± 1.05 ## **	8.89 ± 1.37 † **
Thermal Discomfort (Body)			
Cold	1.0 ± 0.0	1.76 ± 0.63 ##	1.47 ± 0.61
Neutral	1.10 ± 0.22	1.0 ± 0.0 **	1.47 ± 0.67 †
Hot	1.02 ± 0.11	1.78 ± 0.73 ##	1.84 ± 0.83 *
Muscular Discomfort (Arm)			
Cold	1.15 ± 0.3	1.68 ± 0.7 #	2.76 ± 0.7 ††
Neutral	1.05 ± 0.22	1.05 ± 0.22 **	3.13 ± 0.62 ††
Hot	1.02 ± 0.11	1.21 ± 0.53 *	3.76 ± 0.53 †† **

Data are expressed as mean ± SD. * (*p* < 0.05) vs. cold, ** (*p* < 0.01) vs. cold, # (*p* < 0.05) vs. time point pre-water immersion, ## (*p* < 0.01) vs. pre-water immersion, † (*p* < 0.01) vs. time point post-water immersion, †† (*p* < 0.001) vs. post-water immersion.

## Appendix A

**Table A1:** NASA-TLX perceived workload.

Variable	Cold	Neutral	Hot
Mental	7.6 ± 4.1	8.2 ± 3.7	10.7 ± 4.2 **
Physical	11.1 ± 3.2	12.5 ± 2.5	13.8 ± 4.0 **
Temporal	8.4 ± 3.9	10.5 ± 3.4 **	10.9 ± 4.1 *
Performance	5.7 ± 3.9	7.3 ± 3.5	7.8 ± 3.8
Effort	11.5 ± 4.4	13.5 ± 1.8 *	14.4 ± 2.3 **
Frustration	4.3 ± 2.9	5.6 ± 3.4	7.1 ± 4.2 *
Sum	48.9 ± 17.4	57.8 ± 11.1 *	65.1 ± 17.3 **

Data are expressed as mean ± SD. * (*p* < 0.05) vs. cold condition, ** (*p* < 0.01) vs. cold.
